# Combined small cell carcinoma of the sinonasal tract associated with syndrome of inappropriate secretion of antidiuretic hormone: A case report

**DOI:** 10.3892/ol.2014.1882

**Published:** 2014-02-13

**Authors:** MIKIKO KAYAKABE, KATSUMASA TAKAHASHI, TOMOFUMI OKAMIYA, ATSUKI SEGAWA, TETSUNARI OYAMA, KAZUAKI CHIKAMATSU

**Affiliations:** 1 Department of Otolaryngology-Head and Neck Surgery, Gunma University Graduate School of Medicine, Maebashi, Gunma 371-8511, Japan; 2 Department of Diagnostic Pathology, Gunma University Graduate School of Medicine, Maebashi, Gunma 371-8511, Japan

**Keywords:** small cell carcinoma, squamous cell carcinoma, neuroendocrine, sinonasal tract, syndrome of inappropriate secretion of antidiuretic hormone

## Abstract

Combined small cell carcinoma (SmCC) and squamous cell carcinoma (SqCC) is a rare malignant neoplasm in the head and neck. This study presents the first reported case of combined SmCC and SqCC originating from the sinonasal tract accompanied by syndrome of inappropriate secretion of antidiuretic hormone (SIADH). An 80-year-old female presented with a four-week history of right nasal discharge, nasal obstruction and left neck swelling. Imaging studies revealed a tumorous lesion in the maxillary sinus encroaching upon the right nasal cavity and left cervical lymph node (LN) swelling. An incisional biopsy carried out from the right maxillary sinus and LNs resulted in a diagnosis of combined SmCC with SqCC, staged as T4aN2cM0. Clinical examination revealed a sustained increase of antidiuretic hormone, hyponatremia with urinary sodium increase, and serum hypo-osmosis, resulting in SIADH. Water restriction to <1,000 ml/day was effective in improving sodium and osmotic imbalance. Curative treatment for the tumor was not prescribed due to the poor condition of the patient. Palliative treatment was administered and the patient succumbed to cachexia five months after histological diagnosis. The presence of SIADH may have marked implications for the treatment and prognosis of this disease.

## Introduction

Neuroendocrine tumors, which cause syndrome of inappropriate secretion of antidiuretic hormone (SIADH), are potentially detectable in small-cell carcinoma (SmCC) of the lung. Up to 10% of patients with pulmonary neuroendocrine SmCC (1) and 4% of extra-pulmonary neuroendocrine SmCC patients (2) develop SIADH. Ma and Lei reviewed all cases (45 in total) of neuroendocrine SmCC in the nasal cavity or paranasal sinus pathologically diagnosed over the last 40 years. Only two of the 45 cases were reported to be complicated with SIADH (3).

Combined SmCC, which consists of SmCC and another histological type of carcinoma, accounts for only 5% of all SmCCs in the lungs (4). The combination of SmCC and adenocarcinoma is not unusual; several clinical reports exist (5–7). However, the combination of SmCC and squamous cell carcinoma (SqCC) has not been reported previously. This report presents the first known case of combined SmCC and SqCC accompanied by SIADH as a paraneoplastic syndrome. Written informed consent was obtained from the patient.

## Case report

**Figure 1:**
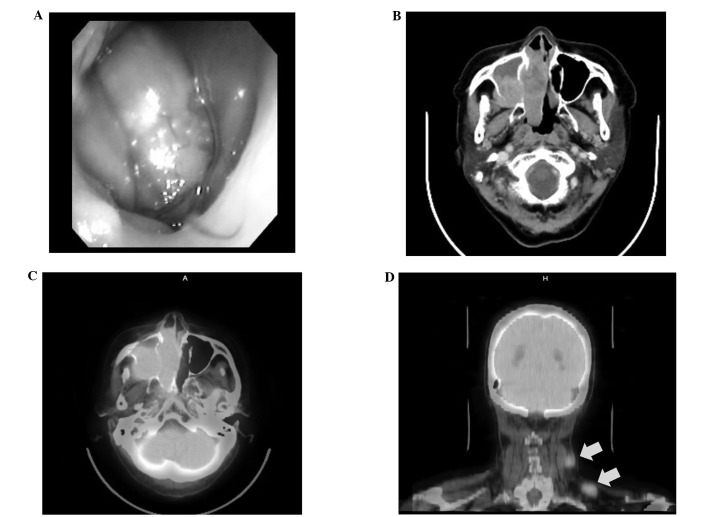
(A) Nasal endoscopic view of an occupying mass in the right nasal cavity. (B) Computed tomography scan revealing the destructive lesion occupying the right maxillary sinus and right nasal cavity with loss of posterior boney walls of the antrum. Fluorodeoxyglucose positron emission tomography revealed high accumulation in (C) the primary tumor and (D) left cervical lymph nodes (arrows).

An 80-year-old Japanese female presented with a four-week history of right nasal discharge, nasal obstruction and left neck swelling. The patient was a non-smoker with a history including left traumatic blindness, chronic renal disorder (estimated glomerular filtration rate on admission, 30.3 ml/min), hypertension and diabetes mellitus. Physical examination demonstrated an easy-bleeding tumor in the right nasal cavity (Fig. 1A) and swelling of the left medial and inferior cervical lymph nodes (diameter, 3 and 2 cm, respectively).

Computed tomography scanning revealed a huge lesion occupying the nasal cavity and paranasal sinus that had destroyed the posterior and medial walls of the maxillary antrum (Fig. 1B). There was direct extension into the right ethmoidal air cells, but no extension to the floor of the orbit, skull base or hard palate. Fluorodeoxyglucose-positron emission tomography (FDG-PET) showed high accumulation in the primary tumor, with a maximum standardized uptake value (SUV_max_) of 9.63 (Fig. 1C). The SUV_max_ of FDG-PET in the left medial and inferior cervical LNs was also high (6.90 and 4.62, respectively; Fig. 1D), although there was no definitive lesion in the right cervical LNs or distant metastasis.

**Figure 2:**
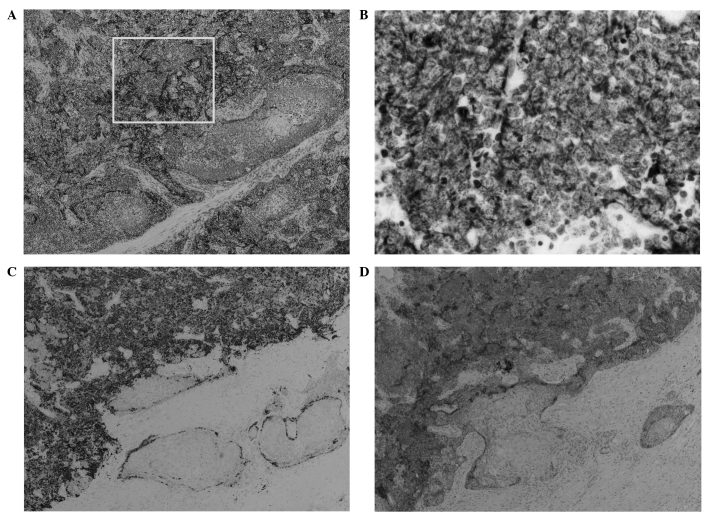
Light microscopy of tumor biopsy from the maxillary sinus. (A) Hematoxylin-eosin staining reveals components of squamous cell carcinoma and sheets of small cells in the marked area. (B) Immunohistochemical staining reveals components of small to intermediate-sized tumor cells positive for CAM5.2. Nuclei have finely stippled or dense chromatin. Components of small-cell carcinoma lesion positive for perinuclear staining with (C) CD56 and (D) synaptophysin. Magnification, (A, C and D) ×40 and (B) ×100.

The tumor in the right nasal cavity was biopsied using forceps, but the pathological examination resulted in necrotic tissue with suppurative granulation. It is unclear whether the biopsy revealed the presence of necrotic tissue or whether the biopsy procedure itself caused the necrotic tissue. An incisional biopsy of the left cervical lymphadenopathy and the right maxillary antral lesion was therefore carried out via the canine fossa. Pathological examination revealed a highly malignant anaplastic tumor. Under hematoxylin-eosin stain, two components could be observed in the lesions: SqCC and small cells containing chromatin-rich nuclei with scanty cytoplasm undergoing apoptosis and mitosis (Fig. 2A). To characterise the cells, an immunohistochemical examination was performed. On evaluation of specimens from the right maxillary sinus, small cells were positive for the epithelial marker cytokeratin CAM5.2 (Fig. 2B), and small cells situated on the peripheral lesion were positive for cluster of differentiation (CD)56 and synaptophysin, neuroendocrine markers (Figs. 2C and D). Malignant lymphoma and malignant melanoma were excluded as CD3, CD20 and human melanoma black 45 stains were negative (data not shown). These findings supported a diagnosis of primary combined neuroendocrine SmCC associated with SqCC of the right maxillary sinus. Staining of the left cervical lymph node produced similar results. Finally, the patient was staged as T4aN2cM0 according to the staging system established by UICC in 2009 (8). Serum levels of neuron-specific enolase (NSE) and pro-gastrin-releasing peptide (pro-GRP), tumor markers of small-cell carcinoma, were also elevated [NSE, 25.8 ng/ml, (normal range, 0–12 ng/ml); pro-GRP, 152.0 pg/ml (normal range, <80.0 pg/ml)].

Following the incisional biopsy, the patient was diagnosed with SIADH on the grounds of low sodium (124 mEq/l) with low plasma osmolarity (271 mOsm/l), high plasma ADH levels (26.2 pg/ml; reference range, 0.3–3.5 pg/ml), high urine sodium (39 mEq/l) and high urine osmolarity (301 mOsm/l)without dehydration. After water restriction to <1,000 ml/day, the sodium imbalance and osmotic status improved. To confirm the neuroendocrine characteristics of this tumor, immunohistochemistry using the rabbit polyclonal anti-human ADH antibody (diluted 1:400; Abcam Inc., Cambridge, UK) was performed; however, the tumor was negative for ADH immunostaining (data not shown). Two weeks after the biopsy, the patient complained of progressive loss of vision in the right eye, induced by tumor extension to the right orbit. Curative treatments, including surgery, irradiation and/or chemotherapy, were not prescribed due to high performance status (grade 3) and severe complicated diseases. Instead, palliative treatments were administered. The patient succumbed to cachexia five months after diagnosis.

## Discussion

Carcinoma of the nasal cavity and paranasal sinus is relatively rare, reported to comprise only 3% of all malignant head and neck diseases (9). Among such sinonasal cancers, SmCC is rarer still, and only 45 cases have been reported (3,5). In addition, there have been no reports of the combination of SmCC and SqCC in the nasal cavity and paranasal sinus.

Combined SmCC is a histological group of carcinomas in which SmCC and non-SmCC, including SqCC or adenocarcinoma, are mixed. In contrast to head and neck cancers, the neuroendocrine type of combined SmCC has been frequently reported in SmCC of the lung. To the best of our knowledge, 17 cases of combined SmCC with SqCC in the larynx (10) and four cases of combined SmCC with adenocarcinoma in the sinonasal tract have been reported (5–7). This is the first report to describe combined SmCC with SqCC in the sinonasal tract.

According to the 2005 WHO pathological classification of tumors of the head and neck (11), neuroendocrine tumors are extremely rare in the sinonasal tract. The neuroendocrine type of SmCC was not described in the chapter on the sinonasal tract, but rather classified under the larynx. The pathology in this case was similar to combined SmCC of the larynx. An important differential diagnosis is olfactory neuroblastoma. SmCC is characterized by the marked appearance of crushed nuclei, which is different to olfactory neuroblastoma. In addition, olfactory neuroblastoma does not coexist with SqCC. Thus, a diagnosis of olfactory neuroblastoma was rejected in favor of combined SmCC.

Hyponatremia due to SIADH is a well known paraneoplastic syndrome and occurs in a variety of malignancies, particularly small-cell lung cancer (11–15%) (1,12) and head and neck cancer (3%) (12,13). Indeed, several reports have demonstrated that the incidence of SIADH in patients with SqCC of the head and neck is markedly higher than previously recognized (13,14). Four mechanisms exist for the overproduction of ADH, namely: i) Ectopic ADH secretion; ii) increased hypothalamic production of ADH-like substances in neurological disorders; iii) administration of drugs, including chemotherapeutic agents; and iv) administration of exogenous ADH. In this case, ADH production from either SmCC or SqCC was not detected by immunohistochemistry, and this patient exhibited neither intracranial invasion nor brain metastasis, and had not received any chemotherapeutic agents. However, serum ADH was elevated (10 times higher than normal) and the clinical data were compatible with a diagnosis of SIADH. These findings imply that the mechanism of SIADH in this patient is unclear, and various factors, including other mechanisms, may be responsible for this pathophysiological state.

Notably, several reports have described that SIADH correlates with clinical factors, including prognosis and the development of metastases. Hansen *et al* demonstrated that, in lung SmCC, hyponatremia is an independent marker of worsening prognosis, in addition to advanced staging, high performance status (≥3), male gender and age >70 years (15). Furthermore, the inability to achieve a normal level of plasma sodium following treatment is also a poor survival indicator. Similarly, in SmCC of the head and neck region, the prognosis of patients with SIADH appears to be poor (16). By contrast, List *et al* revealed that the development of SIADH does not correlate with the clinical stage or distribution of metastatic sites of SmCC (1); thus, the relationship between the presence of SIADH and clinical outcome remains controversial. In the present case report, although the patient recovered immediately from SIADH-induced hyponatremia with water restriction therapy, the tumors progressed rapidly and invaded the right orbit and skull base, and the patient succumbed to the disease five months later. Further data collection from such cases is required to elucidate the pathophysiology of malignancies associated with SIADH.

In conclusion, this report presents the first known case of combined SmCC and SqCC in the sinonasal tract accompanied by SIADH. The presence of SIADH may have marked implications for the treatment and prognosis of this disease. More studies are required to establish the exact cause and appropriate treatments.
